# LPS–TLR4 signaling attenuates CHOP-mediated apoptosis under endoplasmic reticulum stress conditions during porcine embryonic development

**DOI:** 10.3389/fcell.2026.1750233

**Published:** 2026-02-24

**Authors:** Gyu-Hyun Lee, Cheng-Lin Zhan, Song-Hee Lee, Qin-Yue Lu, Ying-Yan Jin, Ji-Yeon Lee, Kyung-Tae Shin, Xiang-Shun Cui

**Affiliations:** Department of Animal Science, Chungbuk National University, Cheongju, Chungbuk, Republic of Korea

**Keywords:** apoptosis, CHOP, endoplasmic reticulum stress, porcine embryonic development, TLR4

## Abstract

**Introduction:**

Persistent endoplasmic reticulum (ER) stress impairs early embryonic development by inducing apoptosis through C/EBP homologous protein (CHOP). Toll-like receptor 4 (TLR4), traditionally recognized for its role in innate immunity, has recently emerged as a modulator of intracellular stress responses. Lipopolysaccharide (LPS), a natural TLR4 agonist derived from Gram-negative bacteria, elicits both pro-inflammatory and cytoprotective effects depending on the cellular context and dosage. This study aimed to elucidate the role of TLR4 signaling in the regulation of CHOP-mediated apoptosis during porcine preimplantation development under ER stress.

**Methods:**

Porcine embryos were treated with tunicamycin (TM, 5 nM) to induce ER stress and co-treated with LPS (10 μM) to activate TLR4 signaling. Developmental competence was assessed by blastocyst formation rates, total cell number, and markers of apoptosis and autophagy.

**Results:**

LPS treatment significantly improved blastocyst formation rates compared to TM groups (TM: 37.50 ± 4.77% vs. TM+LPS: 52.89 ± 4.86%). Consistent with this improvement, the total cell number per blastocyst was significantly restored by LPS co-treatment (Control: 55.63 ± 2.15 vs. TM: 38.61 ± 2.57; TM+LPS: 48.84 ± 0.83), confirming enhanced cell proliferation under ER stress conditions. LPS co-treatment markedly reduced CHOP protein expression and suppressed ATF4 expression, indicating alleviation of PERK-ATF4-CHOP signaling. Additionally, autophagy and apoptosis were attenuated, as evidenced by a significantly decreased LC3-II/LC3-I ratio and a reduced number of TUNEL-positive cells. Notably, TLR4 knockdown abolished these LPS-mediated protective effects, confirming the requirement of TLR4 in mitigating ER stress-induced damage.

**Conclusion:**

These findings demonstrated that LPS-mediated TLR4 signaling suppressed CHOP-induced apoptosis and autophagy under persistent ER stress, thereby improving embryonic viability. This study provides novel mechanistic insights into the non-canonical role of TLR4 in early embryonic development and highlights its therapeutic potential for improving in vitro embryo culture systems.

## 1 Introduction

Embryonic development is a highly orchestrated process that depends on the precise regulation of cell proliferation, differentiation, and survival (Tam and Loebel, 2007). Embryonic development can be adversely affected by stress conditions, such as endoplasmic reticulum (ER) stress, which interfere with essential cellular processes required for proper development (Lin et al., 2019). ER stress arises when the load of misfolded or unfolded proteins exceeds the protein-folding capacity of the ER, triggering the unfolded protein response (UPR), a conserved adaptive mechanism that aims to restore ER function (Wang et al., 2010; Abbadie and Pluquet, 2020) and is active in mammalian preimplantation embryos (Basar et al., 2014).

The UPR comprises three principal signaling branches mediated by kinase R-like endoplasmic reticulum kinase (PERK), activating transcription Factor 6 (ATF6), and inositol-requiring enzyme 1 (IRE1) that mitigate stress by enhancing protein folding capacity and attenuating protein synthesis (Ron and Walter, 2007). However, unresolved or excessive ER stress activates pro-apoptotic signaling pathways, such as C/EBP homologous protein (CHOP), that lead to cell death (Walter and Ron, 2011; Wang and Kaufman, 2012). Among these, the PERK–eukaryotic initiation factor 2 alpha (eIF2α)–ATF4–CHOP axis is a key mediator of ER stress–induced apoptosis, balancing adaptive and apoptotic outcomes depending on the severity and duration of stress (Hu et al., 2018).

In mammalian embryos, ER stress negatively influences developmental competence (Guo et al., 2018; Kim et al., 2024). ER stress reduces blastocyst formation and increases apoptosis, whereas alleviation of ER stress enhances embryo development and reduces apoptotic indices in both mouse (Zhang et al., 2012) and porcine (Park et al., 2018) models. These findings underscore that precise modulation of ER stress and UPR signaling is critical for early embryogenesis.

Recent advances in immunology and molecular biology have highlighted the potential of the Toll-like receptor 4 (TLR4) signaling pathway not only governs innate immune responses but also intersects with cellular stress–response pathways (Tan et al., 2025). Lipopolysaccharide (LPS), a canonical TLR4 agonist, has been extensively studied for its role in innate immunity, particularly in activating downstream signaling cascades through the TLR4–TRIF pathway (Luo et al., 2025). Notably, accumulating evidence indicates that TLR4–TRIF signaling can modulate endoplasmic reticulum (ER) stress outcomes. Specifically, TRIF-dependent signaling has been shown to attenuate ER stress–induced apoptosis by regulating translational control mechanisms, including modulation of eIF2B activity and suppression of CHOP expression (Woo et al., 2012). These findings suggest that LPS-induced TLR4 activation may function as a protective adaptive signal under ER stress conditions, providing a mechanistic basis for exploring its potential role in alleviating ER stress and improving embryonic development competence.

Although previous studies have suggested that LPS–induced TLR4 signaling can suppress CHOP expression under ER stress conditions (Woo et al., 2012), the underlying mechanisms and their developmental relevance during porcine embryogenesis remain largely unexplored. In particular, how TLR4 signaling interfaces with key UPR components—including PERK, ATF4, and the translational regulator eIF2B—and how this interaction influences embryonic stress resilience and cell fate decisions have not been clearly defined.

In this study, we investigated whether LPS modulated ER stress-induced apoptosis in porcine embryos by suppressing CHOP expression via the TLR4 signaling pathway. The key objective of this study was to elucidate the mechanistic link between LPS–TLR4 signaling and UPR-mediated apoptotic regulation under persistent ER stress. By defining the functional relevance of TLR4-dependent stress response modulation during early embryogenesis, this study aims to provide mechanistic insights into strategies that may enhance developmental competence in swine reproduction.

## 2 Materials and methods

### 2.1 Ethics statement

The present study did not involve any live animals. All porcine ovaries used in this study were obtained from a licensed commercial slaughterhouse as by-products of routine meat production. The animals were slaughtered for food purposes and were not sacrificed or handled specifically for research. Therefore, no ethical approval was required for this study in accordance with institutional and national guidelines.

### 2.2 Oocyte collection and *in vitro* maturation (IVM)

Porcine ovaries were collected from a local slaughterhouse (Farm Story Dodram B&F, Umsung, Korea) and transported in saline containing 75 μg/mL penicillin G and 50 μg/mL streptomycin sulfate at 30 °C–37 °C. Cumulus-oocyte complexes (COCs) were aspirated from 3 to 6 mm follicles using an 18-gauge needle. COCs with at least three layers of compact cumulus cells and homogeneous ooplasm were selected and washed in TCM-199 medium (Invitrogen, Carlsbad, CA, US). Approximately 70–100 COCs per well were cultured in four-well plates at 38.5 °C and 5% CO_2_ for 44 h.

### 2.3 Parthenogenetic activation and *in vitro* culture (IVC)

After maturation, cumulus cells were removed using 1 mg/mL hyaluronidase. MII oocytes with a visible polar body were selected and activated by two direct-current pulses of 120 V for 60 μs in an activation buffer consisting of 297 mM mannitol, 0.1 mM CaCl_2_, 0.05 mM MgSO_4_, 0.01% PVA, and 0.5 mM HEPES (pH 7.2). Activated oocytes were incubated in PZM-5 medium with 7.5 μg/mL cytochalasin B for 3 h to prevent extrusion of the second polar body, then washed and cultured in PZM-5 containing 4 mg/mL BSA at 38.5 °C under 5% CO_2_. For experimental treatments, tunicamycin (TM) 5 nM and LPS 10 μM were added to the culture medium immediately after parthenogenetic activation. The experimental groups included control, TM (5 nM), and TM + LPS (5 nM TM and 10 μM LPS). Embryos were continuously cultured from the single-cell stage to the blastocyst stage in the presence of each treatment. Blastocyst formation rate was assessed on day 7.

### 2.4 Lipopolysaccharide and tunicamycin treatment

For experimental treatments, TM (5 nM) and LPS (10 μM) were added to the culture medium immediately after parthenogenetic activation. Experimental groups included control, TM alone (5 nM), LPS alone (10 μM), and TM + LPS (5 nM TM and 10 μM LPS). Embryos were continuously cultured from the single-cell stage to the blastocyst stage in the presence of each treatment. Blastocyst formation rate was assessed on day 7.

### 2.5 *TLR4* dsRNA preparation and microinjection

Double-stranded RNA (dsRNA) targeting was synthesized using T7 promoter-containing primers and the MEGAscript T7 Kit (Thermo Fisher Scientific, Waltham, MA, US). The primer sequences are F: 5′-AGACAGCAATAGCTTCTCCAGC-3′and R: 5′-GCT​AGG​TTT​GTC​TCA​ACG​GC-3’. After RNase and DNase treatment, the dsRNA was purified and diluted to 1,200 ng/μL in RNase-free water and stored at −80 °C. MII oocytes were activated by electrical stimulation and incubated for 3 h in PZM-5 medium containing 7.5 μg/mL cytochalasin B. After washing, 5–10 pL of dsRNA was injected into the cytoplasm of one-cell embryos using a FemtoJet microinjector (Eppendorf, Hamburg, Germany). Control embryos were injected with GFP dsRNA. All embryos were cultured in PZM-5 medium at 38.5 °C with 5% CO_2_.

### 2.6 Immunofluorescence staining

Embryos were washed whit PBS/PVA and fixed with 3.7% paraformaldehyde for 30 min at room temperature. After permeabilization with 0.5% Triton X-100 for 30 min, samples were blocked in 1% BSA for 1 h. Fixation, permeabilization, and antibody incubation were performed with embryos maintained in 96-well plates. Primary antibodies such as phospho-PERK (1:100, 3179S, Cell signaling), rabbit anti- ATF4 (1:100, 10835-1-AP, Proteintech), rabbit anti-CHOP (1:100, SC-166682; Santa Cruz Biotechnology), rabbit anti-phospho-eIF2α (1:100, AP0341; ABclonal), rabbit anti-eIF2α (1:100, A16205; ABclonal), rabbit anti-LC3 (1:100, ab58610; Abcam), and rabbit anti-cleaved caspase-3 (1:100,9664S, Cell signaling) were applied overnight at 4 °C. Samples were washed and incubated with Alexa Fluor-conjugated secondary antibodies such as anti-mouse IgG 568 goat (1:200, A11004, Invitrogen), anti-mouse IgG 488 (1:200, A21202, Invitrogen), goat anti-rabbit IgG 488 (1:200, A11034, Invitrogen), anti-rabbit IgG 546 (1:200, A10040, Invitrogen) for 1 h at 38.5 °C. Embryos were mounted onto glass slides using DAPI-containing mounting medium, covered with a coverslip, and stored at 4 °C until imaging. Imaging was performed using a confocal microscope (Zeiss LSM 710 META, Oberkochen, Germany), and image analysis was conducted using ImageJ software (National Institutes of Health, Bethesda, MD, US).

### 2.7 Quantitative reverse transcription PCR (qRT-PCR)

Total RNA was extracted from the embryos using the Dynabeads mRNA Direct Kit (Thermo Fisher Scientific). First-strand cDNA was synthesized using a ReverTra Ace kit (TOYOBO, Osaka, Japan). qRT-PCR was performed using the SYBR Green Master Mix (Bio-Rad, Hercules, CA, US) on a CFX96 system (Bio-Rad). Gene expression was normalized to GAPDH and calculated using the 2^–ΔΔCt^ method. Primer sequences are listed in Table 1.

**TABLE 1:** List of primers used in this study.

Gene	Primer sequences (5′-3′)
*IRE1*	F: AGGACGTGAGTGACCGGATA R: GCCGCCTTTATAGGTTCGGA
*PERK*	F: AGGACGTGAGTGACCGGATA R: GCCGCCTTTATAGGTTCGGA
*ATF6*	F: AAT GGA TCA CTG AAG CGG CA R: CCT GTT CCA ATA TAC TCA TAG GTC C
*LC3*	F: CCGAACCTTCGAACAGAGAG R: AGGCTTGGTTAGCATTGAGC
*ATG7*	F: AGATTGCCTGGTGGGTGGT R: GGGTGATGCTGGAGGAGTTG
*BCL-XL*	F: CTT ACC TGA ATG ACC ACC TAG AGC R: CCG ACT GAA GAG CGA ACC C
*BAX*	F: AAC ATC GCC CTG TGG ATG AC R: CAC TTA TGG CCC AGA TAG GCA
*TLR4*	F: GAC AGC AAT AGC TTC TCC AGC R: GGT TTG TCT CAA CGG CAA CC
*GAPDH*	F: AAG TTC CAC GGC ACA GTC AAG R: CAC CAG CAT CAC CCC ATT T
*TLR4*-dsRNA	F: TAA TAC GAC TCA CTA TAG GGA AAC CAC TCC ACT CCC TCA G R: TAA TAC GAC TCA CTA TAG GGT TCC TCA CCC AGT CTT CGT C

### 2.8 Protein extraction and western blotting analysis

A total of 80–100 embryos per group were lysed in RIPA buffer with protease/phosphatase inhibitors (Thermo Fisher Scientific) and boiled at 95 °C for 10 min. Proteins were separated by SDS-PAGE and transferred to PVDF membranes. After blocking with 5% skim milk, the membranes were incubated overnight at 4 °C with primary antibodies such as rabbit anti-ATF4 (1:1000, 10835-1-AP, Proteintech), rabbit anti-CHOP (1:1000, SC-166682; Santa Cruz Biotechnology), rabbit anti-phospho-eIF2α (1:1000, AP0341; ABclonal), rabbit anti- LC3 (1:800, ab58610; Abcam), rabbit anti-TLR4 (1:1000, 19811-1-AP, Proteintech), and rabbit anti-GAPDH (1:1000, sc-365062, Santa Cruz Biotechnology). Membranes were then incubated for 1 h with an horseradish peroxidase-conjugated secondary antibody. Signal detection was performed using enhanced chemiluminescence, and imaging was conducted using UVITEC Q9 mini software (Uvitec, Cambridge, United Kingdom).

### 2.9 TUNEL assay

Apoptotic cells in the blastocysts were detected using an *In Situ* Cell Death Detection Kit. Fixed and permeabilized embryos were incubated with TUNEL reaction solution at 37 °C for 1 h, then mounted in DAPI-containing medium. Samples were examined under a confocal microscope, and the apoptotic index was calculated as the percentage of TUNEL-positive nuclei relative to total nuclei.

### 2.10 Statistical analysis

The experiment was repeated at least three times. All graph data are shown as the mean ± standard error of the mean. Statistical significance was set at *p* < 0.05. All calculations were performed using the GraphPad Prism 10 software (GraphPad, San Diego, CA, US).

## 3 Results

### 3.1 LPS treatment improves blastocyst development under ER stress conditions

**FIGURE 1:**
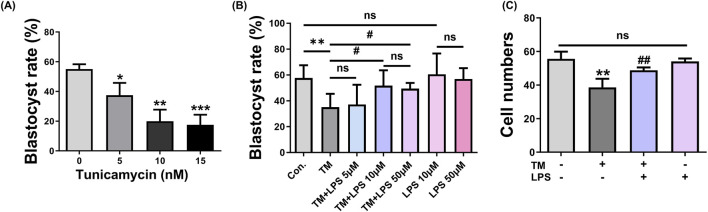
LPS enhances blastocyst formation under ER stress. **(A)** Blastocyst (BL) formation rates of porcine embryos treated with increasing concentrations of tunicamycin (TM). **(B)** BL formation rates in Con, TM (5 μM), TM + 5µM, 10 μM, 50 µM Lipopolysaccharide (LPS), 10 and 50 µM LPS groups. **(C)** Total cell numbers per blastocyst in Con, TM, TM + LPS, and LPS groups. ns: Not significant; *: *p* < 0.05, **: *p* < 0.01, ***: *p* < 0.001, #: *p* < 0.05, ##: *p* < 0.01.

To determine an appropriate ER stress condition, porcine embryos were first cultured in the presence of increasing concentrations of tunicamycin (TM), and blastocyst formation was assessed (Figure 1A). TM treatment caused a dose-dependent reduction in blastocyst formation, with 5 μM TM significantly decreasing the blastocyst rate compared with the control group. (Control: 60.57% ± 9.26% vs. TM: 37.50% ± 4.77%, *p* < 0.05). This concentration was therefore selected for subsequent experiments as a moderate but robust ER stress condition. Next, we assessed whether LPS could ameliorate TM-induced developmental defects and evaluated its concentration-dependent effect (Figure 1B). Treatment with LPS at 5 μM did not significantly improve blastocyst formation compared to the TM group (TM: 35.10% ± 7.38% vs. TM + LPS 5 μM: 37.18% ± 7.38%, *p* = 0.7825), whereas treatment with LPS at 10 μM significantly restored blastocyst development. Increasing the LPS concentration to 50 μM did not result in a significant difference compared to 10 μM LPS. Importantly, embryos treated with either 10 μM or 50 μM LPS alone showed blastocyst formation rates comparable to the control group, indicating that these concentrations of LPS did not adversely affect embryonic development. Based on these results, 10 μM LPS was selected for subsequent experiments. Consistent with these findings, the total cell number per blastocyst was significantly decreased under ER stress (Control: 55.63% ± 2.15% vs. TM: 38.61% ± 2.57%, *p* < 0.01), whereas LPS co-treatment restored cell proliferation to near-control levels (TM: 38.61 ± 2.57 vs. TM + LPS: 48.84 ± 0.83, *p* < 0.05). Treatment with LPS alone did not significantly differ from that of the control (Control: 55.63% ± 2.15% vs. LPS: 54.08% ± 1.25%).

### 3.2 LPS treatment suppresses CHOP expression under ER stress conditions

**FIGURE 2:**
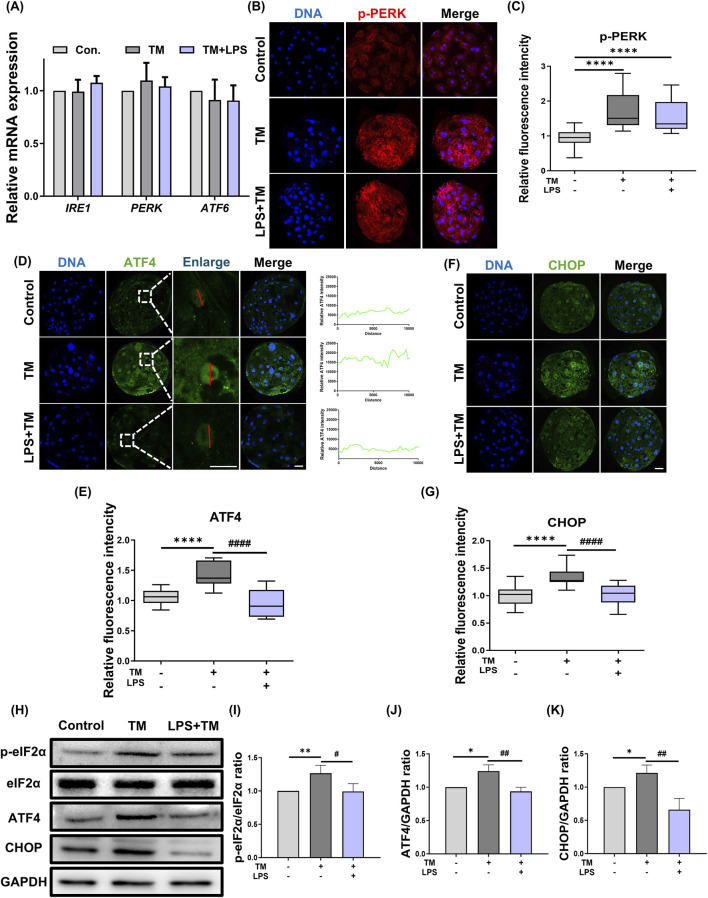
LPS suppresses CHOP expression during ER stress. **(A)** Relative mRNA expression levels of UPR sensors *IRE1*, *PERK*, and *ATF6* in Con, TM, and TM + LPS groups. **(B)** Representative immunofluorescence staining images of p-PERK and DNA in Con, TM, and TM + LPS. **(C)** Quantification of p-PERK fluorescence intensity in Con (*n* = 31), TM (*n* = 30), and TM + LPS (*n* = 33). **(D)** Representative immunofluorescence staining images of ATF4 and DNA in Con, TM, and TM + LPS. **(E)** Quantification of ATF4 fluorescence intensity in Con (*n* = 31), TM (*n* = 35), and TM + LPS (*n* = 33). **(F)** Representative immunofluorescence staining images of CHOP and DNA in Con, TM, and TM + LPS. **(G)** Quantification of CHOP fluorescence intensity in Con (*n* = 30), TM (*n* = 36), and TM + LPS (*n* = 31). **(H)** Western blot images of p-eIF2α, eIF2α, ATF4, CHOP, and GAPDH protein from the 2C to BL stages. GAPDH was used as the internal reference protein. **(I)** Quantification of p-eIF2α protein levels normalized to eIF2α. **(J)** Quantification of ATF4 protein levels normalized to GAPDH. **(K)** Quantification of CHOP protein levels normalized to GAPDH. Scale bar: 20 μm, *: *p* < 0.05, **: *p* < 0.01, ***: *p* < 0.001, ****: *p* < 0.0001, #: *p* < 0.05, ##: *p* < 0.01, ####: *p* < 0.0001.

To investigate whether LPS regulates ER stress-mediated apoptotic signaling, we examined the expression of key proteins in the PERK–ATF4–CHOP pathway using immunofluorescence staining and Western blotting. Quantitative values were normalized to those of the control group and are presented as relative fold changes. Immunofluorescence analysis of p-PERK (Figures 2B,C) revealed that both the TM and TM + LPS groups exhibited increased p-PERK expression compared to the control group (Control: 1.00 ± 0.05 vs. TM: 1.72 ± 0.11, *p* < 0.0001; Control: 1.00 ± 0.05 vs. TM + LPS: 1.49 ± 0.08, *p* < 0.0001). However, the expression levels of the downstream effectors ATF4 and CHOP were significantly decreased upon LPS treatment (ATF4: TM: 1.24 ± 0.05 vs. TM + LPS: 0.93 ± 0.05, *p* < 0.0001; CHOP: TM: 1.21 ± 0.06 vs. TM + LPS: 0.66 ± 0.09, *p* < 0.0001). Western blot analysis showed that TM treatment markedly increased ATF4 and CHOP expression relative to the control (ATF4: Control: 1.00 vs. TM: 1.24 ± 0.05, *p* < 0.05; CHOP: Control: 1.00 vs. TM: 1.21 ± 0.06, *p* < 0.05), whereas the TM + LPS group exhibited a significant reduction in ATF4 and CHOP levels (ATF4: TM: 1.24 ± 0.05 vs. TM + LPS: 0.93 ± 0.05, *p* < 0.01; CHOP: TM: 1.21 ± 0.06 vs. TM + LPS: 0.66 ± 0.09, *p* < 0.01, Figures 2H–K). These results suggest that LPS does not inhibit the upstream activation of PERK, but instead intervenes at the downstream level to block the transcriptional upregulation of ATF4 and CHOP. To further elucidate the mechanism by which LPS attenuates ATF4 and CHOP expression without suppressing PERK phosphorylation, we examined the phosphorylation of eIF2α, a critical intermediate downstream of PERK. Western blot analysis revealed that LPS significantly decreased the level of phosphorylated eIF2α compared to the TM group (TM: 1.26 ± 0.05 vs. TM + LPS: 0.99 ± 0.05, *p* < 0.05), suggesting that LPS acts by restoring eIF2B-mediated translation initiation and preventing the sustained activation of the ATF4–CHOP axis.

### 3.3 LPS treatment reduces ER stress-induced autophagy during porcine embryo development

**FIGURE 3:**
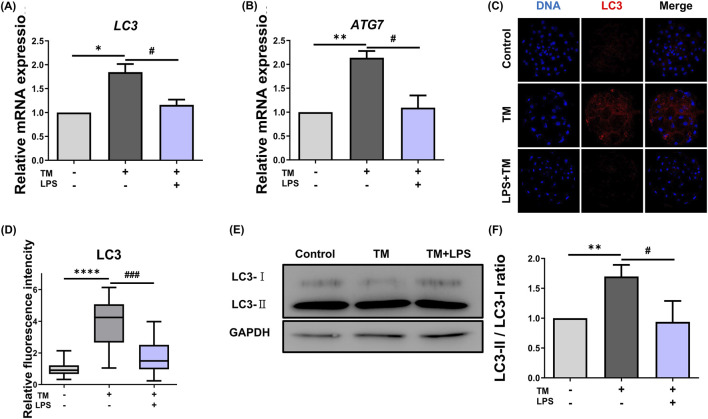
LPS reduces ER stress-induced autophagy in embryos. **(A)** Relative mRNA expression levels of *LC3* in Con, TM, and TM + LPS groups. **(B)** Relative mRNA expression levels of *ATG7* in Con, TM, and TM + LPS groups. **(C)** Representative immunofluorescence staining images of LC3 and DNA in Con, TM, and TM + LPS. **(D)** Quantification of LC3 fluorescence intensity in Con (*n* = 34), TM (*n* = 32), and TM + LPS (*n* = 32). **(E)** Western blot images of LC3-I, LC3-II, and GAPDH protein from the 2C to BL stages. **(F)** Quantification of LC3-II protein levels normalized to LC3-I. Scale bar: 20 μm, *: *p* < 0.05, **: *p* < 0.01, ****: *p* < 0.0001, #: *p* < 0.05, ###: *p* < 0.001.

To examine whether LPS modulated autophagy under persistent ER stress, we assessed the mRNA and protein expression levels of autophagy markers under TM-induced ER stress. qRT-PCR analysis revealed that TM treatment increased the mRNA expression of autophagy-related genes *LC3* (Control: 1.00 vs. TM: 1.84 ± 0.12, *p* < 0.05) and *ATG7* (Control: 1.00 vs. TM: 2.13 ± 0.10, *p* < 0.01) compared to the control. In contrast, LPS co-treatment reduced their transcript levels (*LC3*: TM: 1.84 ± 0.12 vs. TM + LPS: 1.16 ± 0.01, *p* < 0.05; *ATG7*: TM: 2.13 ± 0.10 vs. TM + LPS: 1.09 ± 0.18, *p* < 0.05, Figures 3A,B). This indicates that LPS may attenuate the transcriptional activation of autophagy under ER stress. Consistent with these results, immunofluorescence staining for LC3 protein demonstrated a strong accumulation of LC3 puncta in the TM group compared to the control (Control: 1.00 vs. TM: 3.71 ± 0.49, *p* < 0.0001), indicating elevated autophagic activity. In contrast, the TM + LPS group exhibited markedly reduced LC3 staining intensity (TM: 3.71 ± 0.49 vs. TM + LPS: 1.97 ± 0.27, *p* < 0.001). Western blotting further supported these observations. TM treatment increased the conversion of LC3-I to LC3-II, which is a hallmark of autophagosome formation. In contrast, co-treatment with LPS reduced the LC3-II/LC3-I ratio (Control: 1.00 vs. TM: 1.69 ± 0.11, *p* < 0.01), whereas co-treatment with LPS reduced the LC3-II/LC3-I ratio (TM: 1.69 ± 0.11 vs. TM + LPS: 0.93 ± 0.20, *p* < 0.05, Figures 3E,F).

### 3.4 LPS treatment restores ER stress-induced apoptosis by suppressing pro-apoptotic signaling in porcine embryos

**FIGURE 4:**
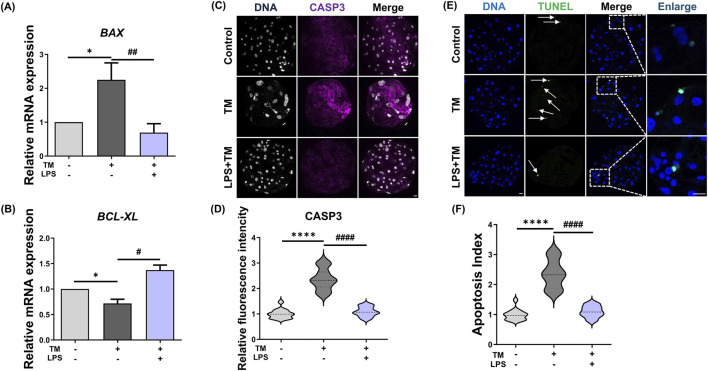
LPS prevents apoptosis caused by ER stress. **(A)** Relative mRNA expression levels of *BAX* in Con, TM, and TM + LPS groups. **(B)** Relative mRNA expression levels of *BCL-XL* in Con, TM, and TM + LPS groups. **(C)** Representative immunofluorescence staining images of CASP3 and DNA in Con, TM, and TM + LPS. **(D)** Quantification of CASP3 fluorescence intensity in Con (*n* = 42), TM (*n* = 34), and TM + LPS (*n* = 50). **(E,F)** TUNEL assay and apoptosis index at the blastocyst stage in Con (*n* = 37), TM (*n* = 32), and TM + LPS (*n* = 36). Scale bar: 20 μm, *: *p* < 0.05, ****: *p* < 0.0001, #: *p* < 0.05, ####: *p* < 0.001.

To determine whether LPS alleviates ER stress-induced apoptosis during porcine embryonic development, we first analyzed the expression of the apoptosis-related genes *BCL-XL* and *BAX* by qRT-PCR. As shown in Figure 4A, TM treatment upregulated the expression of *BAX* (Control: 1.00 vs. TM: 1.94 ± 0.36, *p* < 0.05) and decreased the expression level of the anti-apoptotic gene *BCL-XL* (Control: 1.00 vs. TM: 0.71 ± 0.05, *p* < 0.05). In particular, the increased expression of *BAX* suggests enhanced apoptotic signaling. In contrast, co-treatment with LPS attenuated the TM-induced downregulation of *BAX* (TM: 1.94 ± 0.36 vs. TM + LPS: 0.67 ± 0.10, *p* < 0.05), while *BCL-XL* (TM: 0.71 ± 0.05 vs. TM + LPS: 1.37 ± 0.07, *p* < 0.05) expression remained at a level similar to that of the control, indicating that LPS may shift the balance toward cell survival. Immunofluorescence staining for cleaved Caspase-3 (CASP3), a key executioner of apoptosis, showed strong signal intensity in the TM-treated group, whereas the signal was markedly reduced in the TM + LPS group, like the control (Control: 1.00 ± 0.02 vs. TM: 2.51 ± 0.16, *p* < 0.0001; TM: 2.51 ± 0.1, TM + LPS: 1.08 ± 0.05, *p* < 0.0001, Figures 4C,D). To confirm the anti-apoptotic effect of LPS, a TUNEL assay was performed to detect DNA fragmentation. TM-treated embryos showed a prominent increase in TUNEL-positive nuclei, whereas LPS co-treatment significantly reduced the number of apoptotic cells (Con: 0.98 ± 0.03, vs. TM: 2.35 ± 0.10, *p* < 0.0001; TM: 2.35 ± 0.10 vs. TM + LPS: 1.09 ± 0.03, *p* < 0.0001, Figures 4E,F).

### 3.5 TLR4 knockdown abolishes the protective effect of LPS on ER stress-induced UPR signaling

**FIGURE 5:**
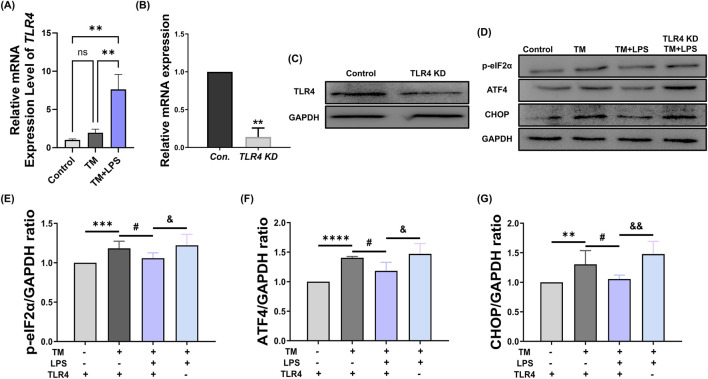
TLR4 knockdown abolishes LPS-mediated UPR regulation. **(A)** Relative mRNA expression levels of TLR4 at the blastocyst stage in the control, TM, and TM + LPS group. **(B)** Relative mRNA expression levels of *TLR4* at the blastocyst stage in the control group (GFP dsRNA) and the *TLR4* KD group. **(C)** Western blot images of TLR4, and GAPDH protein from the 2C to BL stages. **(D)** Western blot images of p-eIF2α, ATF4, CHOP, and GAPDH protein from the 2C to BL stages. **(E)** Quantification of p-eIF2α protein levels normalized to GAPDH. **(F)** Quantification of ATF4 protein levels normalized to GAPDH. **(G)** Quantification of CHOP protein levels normalized to GAPDH. **: *p* < 0.01, ***: *p* < 0.001, ****: *p* < 0.0001, #: *p* < 0.05, and: *p* < 0.05, andand: *p* < 0.01.

The TLR4 signaling pathway modulates cellular stress responses and can be activated by LPS. Therefore, we hypothesize that the alleviating effect of LPS may be achieved through the activation of TLR4. Our results show that TLR4 mRNA expression was significantly increased by LPS (Figure 5A). To investigate whether the recovery effect of LPS on ER stress was mediated by TLR4 signaling, we first confirmed the efficiency of TLR4 knockdown using siRNA. As shown in Figures 5B,C, both mRNA and protein levels of TLR4 were significantly reduced in the TLR4 KD group compared to those in the control, confirming effective gene silencing. We then assessed whether TLR4 knockdown affects the LPS-mediated suppression of the PERK–eIF2α–ATF4–CHOP signaling pathway. As shown in Figures 5D–G, TM treatment increased the levels of p-eIF2α (Control: 1.00 vs. TM: 1.18 ± 0.04, *p* < 0.001), ATF4 (Control: 1.00 vs. TM: 1.40 ± 0.01, *p* < 0.0001), and CHOP (Control: 1.00 vs. TM: 1.30 ± 0.09, *p* < 0.01). In contrast, co-treatment with LPS decreased the expression of all three proteins (p-eIF2α: TM: 1.18 ± 0.04 vs. TM + LPS: 1.05 ± 0.02, *p* < 0.05; ATF4: TM: 1.40 ± 0.01 vs. TM + LPS: 1.18 ± 0.07, *p* < 0.05; CHOP: TM: 1.30 ± 0.09 vs. TM + LPS: 1.05 ± 0.02, *p* < 0.05), a finding consistent with previous studies. However, in the TLR4 KD + TM + LPS group, the expression of p-eIF2α, ATF4, and CHOP proteins was increased to levels comparable to the TM group (p-eIF2α: TM + LPS: 1.05 ± 0.02 vs. TLR4 KD + TM + LPS: 1.22 ± 0.06, *p* < 0.05; ATF4: TM + LPS: 1.18 ± 0.07 vs. TLR4 KD + TM + LPS: 1.47 ± 0.08, *p* < 0.05; CHOP: TM + LPS: 1.05 ± 0.02 vs. TLR4 KD + TM + LPS: 1.47 ± 0.08, *p* < 0.01), indicating a loss of the LPS-mediated suppressive effect.

## 4 Discussion

In this study, we demonstrated that LPS reduced apoptosis in porcine embryos under persistent ER stress via TLR4 signaling. Normally, sustained activation of the PERK–eIF2α–ATF4–CHOP pathway promotes pro-apoptotic signaling and compromises embryonic development (Chai et al., 2025). Our findings revealed that LPS did not block PERK phosphorylation but decreased eIF2α phosphorylation, leading to reduced ATF4 and CHOP expression. This suggests that TLR4 signaling selectively modulates the downstream UPR components to alleviate ER stress-induced apoptosis without triggering nonspecific immune activation or toxicity. Together, these results indicate that LPS selectively modulates the PERK–eIF2α–ATF4–CHOP axis downstream of PERK activation, thereby alleviating ER stress–induced apoptosis without broadly suppressing UPR signaling.

Under conditions of unresolved ER stress, cells activate UPR to restore proteostasis (Hetz, 2012). Although the UPR encompasses three primary branches, PERK, IRE1, and ATF6, this study focused on the PERK-eIF2α-ATF4-CHOP axis due to its direct role in translational control and apoptosis induction. (Pakos-Zebrucka et al., 2016). In contrast, the IRE1 and ATF6 pathways predominantly regulate adaptive responses, including chaperone expression and ER-associated degradation (ERAD) (Shoulders et al., 2013). Activated PERK phosphorylates eIF2α, which leads to the selective translation of ATF4 and the subsequent upregulation of CHOP, a key pro-apoptotic factor (Costa-Mattioli and Walter, 2020). Sustained activation of this PERK–eIF2α–ATF4–CHOP axis promotes apoptosis, particularly in contexts such as embryonic development, where cellular stress responses are tightly regulated (Ron and Walter, 2007). Collectively, these findings support the use of 10 μM LPS as an experimental concentration that confers cytoprotective effects under ER stress without inducing inflammatory or toxic responses during porcine embryonic development.

While numerous studies have focused on alleviating ER stress using chemical chaperones or antioxidants such as 4-PBA or TUDCA, these molecules act broadly and often lack specificity in targeting defined signaling nodes within the UPR (Ozcan et al., 2006; Back and Kaufman, 2012; Hetz, 2012). In contrast, although LPS has long been recognized as a pro-inflammatory molecule that activates immune responses via TLR4, a previous study demonstrated its potential to exert protective effects under specific stress conditions (Akira and Takeda, 2004; Woo et al., 2012). Specifically, activation of TLR4 signaling has been shown to suppress ER stress-induced CHOP expression and translational inhibition by modulating eIF2α downstream regulators (Woo et al., 2012). Based on these mechanistic insights, we hypothesized that LPS attenuates the PERK–ATF4–CHOP pathway under continuous ER stress during porcine embryonic development. Consistent with this hypothesis, LPS treatment did not significantly inhibit PERK phosphorylation but resulted in a marked reduction in eIF2α phosphorylation, which was accompanied by decreased expression of ATF4 and CHOP (Figures 2A–L). These findings suggest that LPS acts downstream of PERK, alleviating ER stress responses and contributing to the recovery of cellular homeostasis. Furthermore, the absence of significant differences in the blastocyst rate and total cell number between the control and LPS-only groups suggested that the LPS concentration used in this study did not elicit a strong inflammatory response. Based on these observations, a fixed concentration of 10 μM LPS was used for subsequent experiments in this study. This concentration did not adversely affect embryonic development when applied alone, and was sufficient to elicit a protective effect under TM-induced ER stress. Importantly, increasing the LPS concentration to 50 μM did not result in a further improvement or impairment of blastocyst formation compared to 10 μM, indicating that the protective effect of LPS was not dose-dependent within the tested range. Therefore, 10 μM LPS was selected as a standardized concentration to facilitate mechanistic analyses without introducing additional variability associated with dose escalation. This supports the notion that the observed protective effects were not due to cytotoxicity or immune activation but rather a specific modulation of the ER stress signaling pathway through TLR4. Thus, the coordinated downregulation of autophagy- and apoptosis-related pathways by LPS suggests a global reduction in the cellular stress burden, contributing to improved embryonic survival under prolonged ER stress.

Autophagy is a fundamental cellular mechanism that maintains homeostasis by degrading and recycling intracellular constituents via lysosomal pathways under nutrient deprivation or stress (Mizushima and Komatsu, 2011). During ER stress, autophagy is activated as an adaptive response to alleviate proteotoxic stress and support cell survival (Yorimitsu and Klionsky, 2007). Notably, the PERK–eIF2α–ATF4 signaling axis, a core branch of the UPR, has been shown to induce several autophagy-related genes, including ATG7 and LC3B transcriptionally (Figures 3A–E). In the present study, treatment with TM significantly upregulated the expression of the autophagy markers LC3B and ATG7 in porcine embryos, indicating robust autophagic activation in response to sustained ER stress. Interestingly, co-treatment with LPS resulted in a marked reduction in the expression of these autophagy-related markers compared to that in the TM group, suggesting that LPS alleviates ER stress intensity and thus reduces the cellular demand for autophagic compensation. Thus, the downregulation of LC3B and ATG7 upon LPS co-treatment may reflect the partial restoration of ER function and reduced cellular stress burden. ER stress also interacts with mitochondrial apoptotic signaling, where pro-apoptotic proteins such as BAX and BAK are activated, leading to mitochondrial outer membrane permeabilization and caspase-9 activation (Scorrano et al., 2003). Our findings that LPS reduces BAX expression and Caspase-3 activation suggest its potential role in preserving mitochondrial integrity. The balance between anti-apoptotic proteins, such as BCL-2 and BCL-XL, and pro-apoptotic proteins, such as BAX and BAK, determines cell fate during preimplantation development (Qian et al., 2022). Consistent with previous reports highlighting that the balance between anti-apoptotic and pro-apoptotic proteins determines cell fate (Hardwick and Soane, 2013), our data suggest that LPS shifts this balance toward cell survival, thereby protecting embryonic cells from ER stress-induced apoptosis.

**FIGURE 6:**
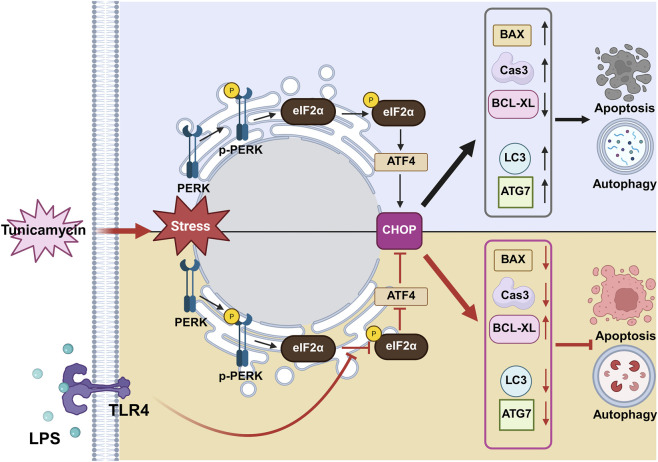
Schematic diagram illustrating the role of TLR4 in signaling CHOP-mediated apoptosis and autophagy during porcine embryonic development under ER stress conditions. During embryonic development, tunicamycin-induced ER stress activates the PERK–eIF2α–ATF4–CHOP signaling axis, which upregulates pro-apoptotic factors (*BAX*, cleaved Caspase-3) and autophagy-related genes (*LC3*, *ATG7*), while downregulating anti-apoptotic *BCL-XL*, ultimately leading to impaired development. Activation of TLR4 by LPS attenuates ER stress signaling by reducing eIF2α phosphorylation and CHOP expression. This suppression shifts the balance toward cell survival by decreasing apoptosis and autophagy, thereby improving embryonic developmental competence under persistent ER stress. Figure created with BioRender.com.

Collectively, these findings suggest that LPS mitigates prolonged ER stress-induced apoptosis in porcine embryos by suppressing CHOP expression, a key pro-apoptotic factor, through TLR4-mediated inhibition of eIF2α phosphorylation. This selective modulation of the PERK–eIF2α–ATF4–CHOP signaling axis attenuates proteotoxic stress and downstream autophagy–apoptosis pathways, thereby contributing to the restoration of cellular homeostasis and supporting embryonic developmental competence under persistent stress conditions. As summarized in Figure 6, the proposed model illustrates how TLR4 activation by LPS rebalances intracellular stress signaling by decreasing phosphorylation of eIF2α, downregulating CHOP-mediated apoptotic and autophagic pathways, and shifting the cellular state toward survival and development. This mechanistic insight not only reveals a novel cytoprotective role of TLR4 signaling in embryogenesis but also highlights its potential utility in improving *in vitro* embryo culture systems under stress-prone conditions. Despite the strengths of this study, it has several limitations. First, LPS is classically recognized as a potent pro-inflammatory molecule, and its effects are highly context-dependent. Although our results demonstrated a protective role of LPS in embryonic survival and development under persistent ER stress, this study did not comprehensively assess inflammatory signaling or cytokine responses across different developmental stages or treatment durations. In addition, this study focused on specific concentrations and exposure windows of LPS during early embryonic development. Therefore, the potential dose-dependent or stage-specific inflammatory effects of LPS have not been fully elucidated. Further studies examining broader inflammatory markers and varying temporal exposure conditions are required to fully characterize the dual roles of LPS in embryonic development. Nonetheless, within the experimental framework used in this study, LPS did not exert overt detrimental effects on embryonic development but instead conferred cytoprotective effects under ER stress (Page et al., 2022).

## Data Availability

The datasets presented in this study can be found in online repositories. The names of the repository/repositories and accession number(s) can be found in the article/supplementary material.

## References

[B1] Abbadie C. Pluquet O. (2020). Unfolded protein response (UPR) controls major senescence hallmarks. Trends Biochem. Sci. 45, 371–374. 10.1016/j.tibs.2020.02.005 32311331

[B2] Akira S. Takeda K. (2004). Toll-like receptor signalling. Nat. Rev. Immunol. 4, 499–511. 10.1038/nri1391 15229469

[B3] Back S. H. Kaufman R. J. (2012). Endoplasmic reticulum stress and type 2 diabetes. Annu. Rev. Biochem. 81, 767–793. 10.1146/annurev-biochem-072909-095555 22443930 PMC3684428

[B4] Basar M. Bozkurt I. Guzeloglu-Kayisli O. Sozen B. Tekmen I. Schatz F. (2014). Unfolded protein response prevents blastocyst formation during preimplantation embryo development *in vitro* . Fertil. Steril. 102, 1777–1784. 10.1016/j.fertnstert.2014.09.004 25305729

[B5] Chai H. Hu Q. Yao S. Ma S. Su W. (2025). Endoplasmic reticulum stress-mediated programmed cell death in the tumor microenvironment. Cell. Death Discov. 11, 559. 10.1038/s41420-025-02862-6 41407677 PMC12712068

[B6] Costa-Mattioli M. Walter P. (2020). The integrated stress response: from mechanism to disease. Science 368, eaat5314. 10.1126/science.aat5314 32327570 PMC8997189

[B7] Guo J. Niu Y. J. Shin K. T. Kwon J. W. Kim N. H. Cui X. S. (2018). Fatty acid synthase knockout impairs early embryonic development via induction of endoplasmic reticulum stress in pigs. J. Cell. Physiol. 233, 4225–4234. 10.1002/jcp.26241 29058795

[B8] Hardwick J. M. Soane L. (2013). Multiple functions of BCL-2 family proteins. Cold Spring Harb. Perspect. Biol. 5. 10.1101/cshperspect.a008722 23378584 PMC3552500

[B9] Hetz C. (2012). The unfolded protein response: controlling cell fate decisions under ER stress and beyond. Nat. Rev. Mol. Cell. Biol. 13, 89–102. 10.1038/nrm3270 22251901

[B10] Hu H. Tian M. Ding C. Yu S. (2018). The C/EBP homologous protein (CHOP) transcription factor functions in endoplasmic reticulum stress-induced apoptosis and microbial infection. Front. Immunol. 9, 3083. 10.3389/fimmu.2018.03083 30662442 PMC6328441

[B11] Kim Y. W. Yang S. G. Seo B. B. Koo D. B. Park H. J. (2024). Deoxynivalenol leads to endoplasmic reticulum stress-mediated apoptosis via the IRE1/JNK/CHOP pathways in porcine embryos. Food Chem. Toxicol. 188, 114633. 10.1016/j.fct.2024.114633 38608924

[B12] Lin T. Lee J. E. Kang J. W. Shin H. Y. Lee J. B. Jin D. I. (2019). Endoplasmic reticulum (ER) stress and unfolded protein response (UPR) in mammalian oocyte maturation and preimplantation embryo development. Int. J. Mol. Sci. 20. 10.3390/ijms20020409 30669355 PMC6359168

[B13] Luo R. Yao Y. Chen Z. Sun X. (2025). An examination of the LPS-TLR4 immune response through the analysis of molecular structures and protein–protein interactions. Cell. Commun. Signal. 23, 142. 10.1186/s12964-025-02149-4 40102851 PMC11921546

[B14] Mizushima N. Komatsu M. (2011). Autophagy: renovation of cells and tissues. Cell. 147, 728–741. 10.1016/j.cell.2011.10.026 22078875

[B15] Ozcan U. Yilmaz E. Ozcan L. Furuhashi M. Vaillancourt E. Smith R. O. (2006). Chemical chaperones reduce ER stress and restore glucose homeostasis in a mouse model of type 2 diabetes. Science 313, 1137–1140. 10.1126/science.1128294 16931765 PMC4741373

[B16] Page M. J. Kell D. B. Pretorius E. (2022). “The role of lipopolysaccharide-induced cell signalling in chronic inflammation,”Chronic Stress. 6. 10.1177/24705470221076390 35155966 PMC8829728

[B17] Pakos‐Zebrucka K. Koryga I. Mnich K. Ljujic M. Samali A. Gorman A. M. (2016). The integrated stress response. EMBO reports 17, 1374–1395. 10.15252/embr.201642195 27629041 PMC5048378

[B18] Park H. J. Park J. Y. Kim J. W. Yang S. G. Jung J. M. Kim M. J. (2018). Melatonin improves the meiotic maturation of porcine oocytes by reducing endoplasmic reticulum stress during *in vitro* maturation. J. Pineal Res. 64. 10.1111/jpi.12458 29149522 PMC5814851

[B19] Qian S. Wei Z. Yang W. Huang J. Yang Y. Wang J. (2022). The role of BCL-2 family proteins in regulating apoptosis and cancer therapy. Front. Oncol. 12, 985363. 10.3389/fonc.2022.985363 36313628 PMC9597512

[B20] Ron D. Walter P. (2007). Signal integration in the endoplasmic reticulum unfolded protein response. Nat. Rev. Mol. Cell. Biol. 8, 519–529. 10.1038/nrm2199 17565364

[B21] Scorrano L. Oakes S. A. Opferman J. T. Cheng E. H. Sorcinelli M. D. Pozzan T. (2003). BAX and BAK regulation of endoplasmic reticulum Ca2+: a control point for apoptosis. Science 300, 135–139. 10.1126/science.1081208 12624178

[B22] Shoulders M. D. Ryno L. M. Genereux J. C. Moresco J. J. Tu P. G. Wu C. (2013). Stress-independent activation of XBP1s and/or ATF6 reveals three functionally diverse ER proteostasis environments. Cell. Rep. 3, 1279–1292. 10.1016/j.celrep.2013.03.024 23583182 PMC3754422

[B23] Tam P. P. Loebel D. A. (2007). Gene function in mouse embryogenesis: get set for gastrulation. Nat. Rev. Genet. 8, 368–381. 10.1038/nrg2084 17387317

[B24] Tan L. Li J. Sun D. Tian X. Zhong X. Shan Y. (2025). TLR4 as a therapeutic target: antidepressant mechanism of saikosaponin A in regulating the NF-κB/BDNF axis and mitigating oxidative stress and inflammation *in vivo* and *in vitro* . Front. Pharmacol. 16, 1585290. 10.3389/fphar.2025.1585290 40453654 PMC12122742

[B25] Walter P. Ron D. (2011). The unfolded protein response: from stress pathway to homeostatic regulation. Science 334, 1081–1086. 10.1126/science.1209038 22116877

[B26] Wang S. Kaufman R. J. (2012). The impact of the unfolded protein response on human disease. J. Cell. Biol. 197, 857–867. 10.1083/jcb.201110131 22733998 PMC3384412

[B27] Wang G. Yang Z. Q. Zhang K. (2010). Endoplasmic reticulum stress response in cancer: molecular mechanism and therapeutic potential. Am. J. Transl. Res. 2, 65–74. 20182583 PMC2826823

[B28] Woo C. W. Kutzler L. Kimball S. R. Tabas I. (2012). Toll-like receptor activation suppresses ER stress factor CHOP and translation inhibition through activation of eIF2B. Nat. Cell. Biol. 14, 192–200. 10.1038/ncb2408 22231169 PMC3271190

[B29] Yorimitsu T. Klionsky D. J. (2007). Endoplasmic reticulum stress: a new pathway to induce autophagy. Autophagy 3, 160–162. 10.4161/auto.3653 17204854

[B30] Zhang J. Y. Diao Y. F. Kim H. R. Jin D. I. (2012). Inhibition of endoplasmic reticulum stress improves mouse embryo development. PLoS One 7, e40433. 10.1371/journal.pone.0040433 22808162 PMC3396646

